# From the challenge of assessing autonomy to the instruments used in practice: A scoping review

**DOI:** 10.1097/j.pbj.0000000000000153

**Published:** 2022-09-09

**Authors:** Andreia Maria Novo Lima, Maria Manuela Ferreira da Silva Martins, Maria Salomé Martins Ferreira, Carla Sílvia Fernandes, Soraia Dornelles Schoeller, Vítor Sérgio Oliveira Parola

**Affiliations:** a Abel Salazar Institute of Biomedical Sciences, Higher School of Health Fernando Pessoa, CINTESIS; Polytechnic Institute of Viana do Castelo, UICISA:E; Parola, Higher School of Health Fernando Pessoa, Porto, Portugal. Health Sciences Research Unit: Nursing (UICISA: E), Nursing School of Coimbra (ESEnfC), Portugal. Portugal Centre for Evidence-Based Practice: A Joanna Briggs Institute Centre of Excellence,; b Nursing School of Porto - CINTESIS, Porto,; c Instituto Politécnico de Viana do Castelo, Viana do Castelo, Portugal,; d Federal University of Santa Catarina, Florianopolis, Brazil,; e University Fernando Pessoa, Health Sciences School, Porto, Portugal.

**Keywords:** personal autonomy, relational autonomy, weights and measures

## Abstract

Autonomy is one of the essential components to live a quality life. Monitoring this autonomy is, in effect, essential, to allow the nurses to conceive, implement and evaluate interventions aimed at its promotion or even maintenance. For this reason, this scoping review aims to map the evidence to identify and analyze the instruments used to assess the person’s autonomy, which emerges from scientific production.

**Methods:**

Scoping review based on the recommended principles by the Joanna Briggs Institute. The research was realized in the databases: Scopus (excluding MEDLINE), CINAHL complete (via EBSCO, Excluding MEDLINE), and MEDLINE (via PubMed). Two independent reviewers evaluated the articles’ pertinence for the study’s investigation, the extraction, and synthesis of articles.

**Results:**

After the analysis, according to the inclusion criteria established, 34 articles were selected, allude to 7 different instruments to assess autonomy.

**Conclusions:**

The need for further development at this level is highlighted, namely through the construction and validation of more comprehensive instruments, integrating the different components of the concept of autonomy.

## Introduction

Autonomy is a multidimensional concept that congregates several attributes, such as cognitive capacity, intellectual capacity, emotional intelligence, social situation, and physical capacity.^1^

Some authors associate autonomy with the ability to consent something, namely invasive health care procedures.^2^ Thus, they consider that autonomous decisions are competent; insofar as they are intentional, they are made based on the understanding and without controlling influences^3^ of third parties. Health empowerment is the foundation of understanding. It is of great relevance, especially concerning the promotion of autonomy, as it allows the development of intellectual capacity, the management of emotions, adaptation to the new physical condition, and social involvement.^4,5^

Other authors associate autonomy only with the functional capacity to carry out daily tasks.^6^ This concept presupposes the capacity to realize daily life’s instrumental activities, decision-making ability, the ability to acquire new knowledge and insight, and motivation for work.^7^ So, we can say that the concept of autonomy is comprehensive, which can induce its users to lessen some of its particularities since it can be interpreted in several ways.^3^

In clinical practice, we daily assist to an imprecise application of the concept of autonomy, when, in fact, they want to refer to the independence of the physical-functional person.^8^ Concerning nursing, it is also noted that, in the care processes, autonomy is, in a way, neglected, with care directed only to the person’s physical-functional independence in daily life activities, designating this type of interventions as promoters of autonomy.^9^ In health-disease processes, the person is losing their autonomy. It is necessary to obtain positive feedback from the health professional and needed support relative to these oscillations,^10^ since hospitalization, for several reasons, namely bureaucratic issues and organizational aspects, promotes changes in dependence at various levels of a person’s life.

In short, the concept of autonomy is a multidimensional concept that encompasses several dimensions of the human being, such as the biological, social, psychological, and spiritual dimension.^11^ In practice, these dimensions are, therefore, represented by the cognitive state, emotional intelligence, social situation, intellectual and physical condition.^1^

As Watson points out, the nurse as a professional, knowledge holder capable of meeting the person’s needs is his function to understand everything that involves and surrounds autonomy,^12^ for its implementation, effectiveness, and monitoring.

Scoping reviews are used to map the key concepts that support the field that is intended to be researched and how to define the conceptual limits of the topics under study. Besides, these studies are useful for examining the available evidence and summarizing the knowledge, giving answers, more specific and valuable to understand a particular subject or discipline.^13^ Mapping the evidence to identify and analyze the instruments used to assess the person’s autonomy can be an excellent contribution to know the instruments and the potential need to build a new instrument, which respond to all areas identified in the concept of autonomy found in this scoping, as well as identifying gaps in the evidence.

Aware of this need and using a scoping review, we intend to map the evidence to identify and analyze the instruments used to assess the person’s autonomy resulting from scientific production.

## Methods

The search for scientific evidence using systematic review is at the heart of evidence-based practice^14^ in most scientific areas, namely in nursing. We opted for a scoping review. It is an investigation methodology whose main objectives are to map the existing implicit evidence to an investigation area and identify the current evidence’s deficiencies.^15^

A scoping review sought to evaluate the existing evidence and learn about the professionals’ instruments to assess the person’s autonomy. The present study intended to make groups, in an organized and systematic way, of the evaluation instruments of the person’s autonomy. This research followed all of the recommended phases by the Joanna Briggs Institute.^13^

Bearing in mind the knowledge to be synthesized, the review had as a starting point the following question: “What assessment instruments are used to assess the person’s autonomy?”.

Using the participants, concept and context strategy (PCC), there were included in the scoping review studies that: (a) according to the type of participants, approach the person (all ages included); (b) according to the concept, approach autonomy; (c) according to the context, use instruments to evaluate autonomy (all contexts); (d) according to the kind of studies, will be completed qualitative and quantitative studies.

The research strategy includes published studies, being performed in 3 steps: (1) Initial research in the databases Scopus, MEDLINE® *(Medical Literature Analysis and Retrieval System Online)* via PubMed, and CINAHL (Cumulative Index to Nursing and Allied Health Literature) via EBSCO, taking place, followed by a text word analysis in the titles and abstracts and the index terms used in the article description; (2) Second research with the resource to the keywords and identified index terms, in the databases included (Table 1); (3) The bibliographic references of the identified articles were examined, to identify additional studies. Studies written in English, Spanish, and Portuguese were considered for inclusion in this review, regardless of publication year (between 2010 until April 18, 2020). Due to the amount of existing primary information, it was considered that the most appropriate methodology was the studies published in the last ten years. It is important to note that there is a greater probability of finding instruments that best suit the reality of the current world in this time period, and this factor was also crucial for the temporal decision. It was also considered the importance of the reviews to include relevant research results and published recently.^16,17^

**Table 1:** Research strategy applied by database and the respective search results by database

Database: Scopus
Filters: Excluding MEDLINE
Results: 813
Search strategy (18 de abril de 2020)
((TITLE-ABS-KEY (patient*)) AND (TITLE-ABS-KEY (independence) OR TITLE-ABS-KEY (autonomy)) AND ((TITLE-ABS-KEY (theory) OR TITLE-ABS-KEY (concept)))) AND NOT (PMID (1*) OR PMID (2*) OR PMID (3*) OR PMID (4*) OR PMID (5*) OR PMID (6*) OR PMID (7*) OR PMID (8*) OR PMID (9*))
Database: CINAHL complete (via EBSCO)
Filters: Excluding MEDLINE
Results: 313
Search strategy (18 de abril de 2020)
S1—MH Patients OR TI patient* OR AB patient*
S2—MH Patient Autonomy OR TI independence OR AB independence
S3—TI theory OR AB theory OR TI concept OR AB concept
S1 AND S2 AND S3
Database: MEDLINE (VIA PUBMED)
Results: 461
Search strategy (18 de abril de 2020)

(((Patients[MeSH Terms]) OR (patient^*^[Title/Abstract])) AND (((Independent Living[MeSH Terms]) OR (Personal Autonomy[MeSH Terms])) OR (independence[Title/Abstract]))) AND ((concept[Title/Abstract]) OR (theory[Title/Abstract])) Filters: in the last 10 years, English, Portuguese, Spanish, MEDLINE

The article relevance for the review was analyzed for 2 independent reviewers, using the title and abstract. The complete articles were subsequently recovered after meeting the inclusion criteria. Independently, 2 reviewers analyzed the articles in fulltext to analyze whether they met the inclusion criteria defined. When there were differences of opinion between the 2 reviewers, a third reviewer’s intervention was requested. In line with the objectives, the researchers developed an instrument, with the starting point, for data extraction.

The following information was mentioned for each study: (a) author, year of publication and country; (b) methodological design; (c) characteristics of the participants; (d) name of the instruments; and (e) objective(s) of the study.

**Figure 1.**
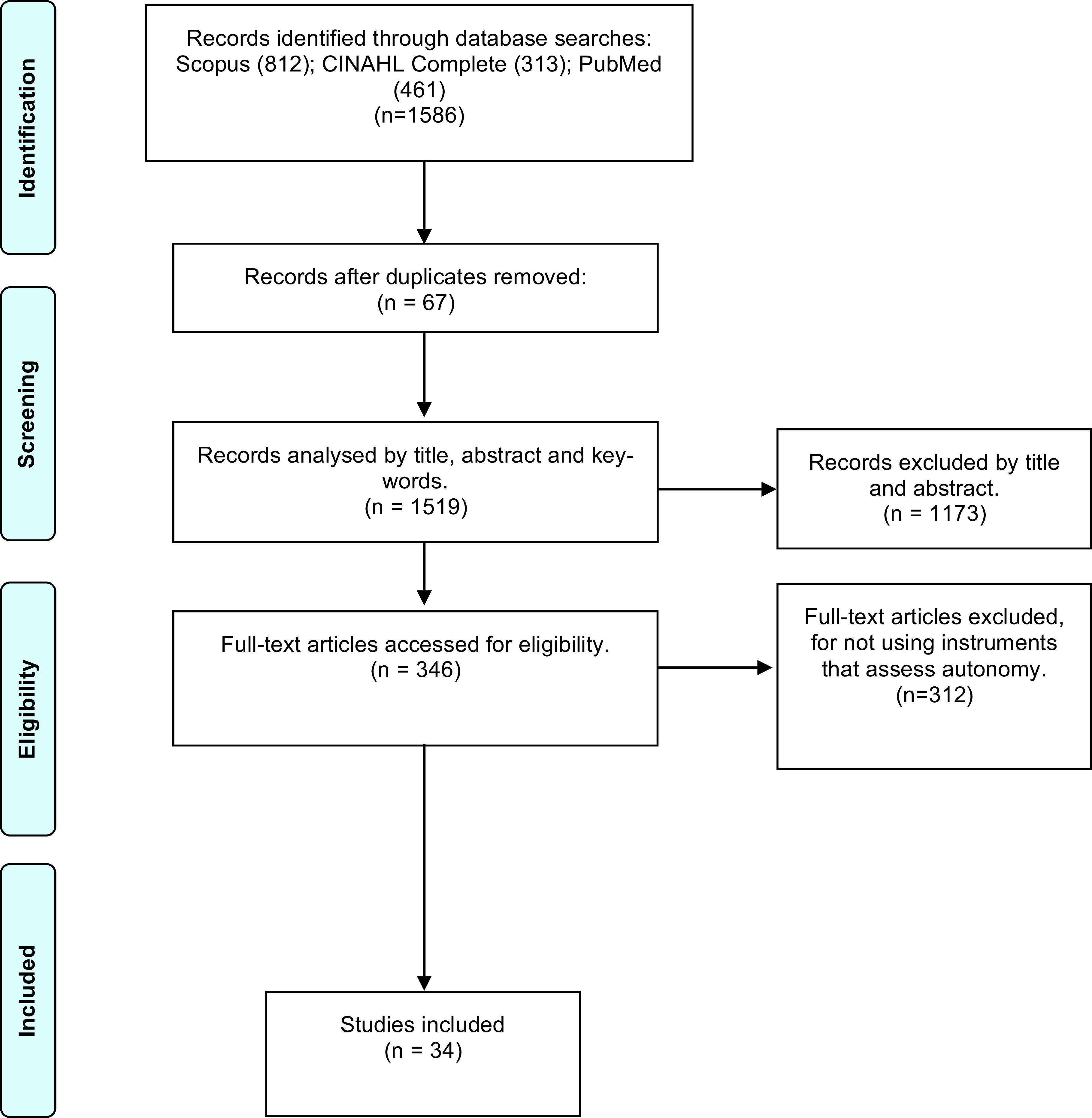
PRISMA flowchart (adapted) of the study selection process.

As shown in Figure 1, the researcher identified 1486 articles with potential pertinence for the presented study. Of these, 67 were extracted because they were in duplicate. Of the remaining 1519 articles, 1173 articles were excluded after reading the title and the abstract and 312 articles were excluded for not meeting the inclusion criteria after full reading. After that selection, 34 articles were included.

## Results

The 34 articles included in the sample are presented in Table 2, completing the following information: study code, authors, kind of study, year, population, instruments used to evaluate autonomy and objectives or study hypotheses.

**Table 2:** Articles in analyses. Porto, Portugal, 2020

A1 (Chen, Chang, Tsai, & Hou, 2018)	Quantitative—correlational and cross-sectional	Effects of perceived autonomy support and basic need satisfaction on quality of life in hemodialysis patients	2018	250 patients in hemodialysis, Taiwan	The Health Care Climate Questionnaire (HCCQ)	Examine whether HD patient’ perceived autonomy support from health care practitioners, including physicians and nurses, relates to their satisfaction of basic needs and in turn influences their HRQOL
A2 (Halvari et al, 2017)	Quantitative—RCT (randomized control trial)	Physical activity and motivational predictors of changes in health behavior and health among DM2 and CAD patients	2017	108 patients with diabetes mellitus type 2 and coronary artery disease, Norway	Treatment Self-Regulation Scale (ΓSRQ)	To test if the SDT model for health behavior can account for change in glucose control and other health outcomes
A3 (Farholm, Halvari, Niemiec, Williams, & Deci, 2017)	Quantitative—correlational and longitudinal	Changes in return to work among patients in vocational rehabilitation: a self-determination theory perspective	2016	90 patients with musculoskeletal disorders and/or light-to-moderate mental disorders, Norway	HCCQ TSRQ	To examine whether patient perceptions of autonomy support from the treatment team in an avocational rehabilitation program will be associated with change (increase) in need satisfaction, autonomous motivation, perceived competence, well-being, physical activity, and return to work, and whether the self-determination theory Model of Behavior will provide an adequate fit to the data
A4 (Jochems, Duivenvoorden, van Dam, van der Feltz-Cornelis, & Mulder, 2017)	Quantitative—cluster randomized control trial (RCT)	Motivation, treatment engagement and psychosocial outcomes in outpatients with severe mental illness: a test of Self-Determination Theory	2015	294 patients and their 57 clinicians, Netherlands	HCCQ	Investigate the basic process model of SDT in outpatients with severe mental illness
A5 (Jochems, Mulder, Duivenvoorden, van der Feltz-Cornelis, & van Dam, 2014)	Quantitative—scale translation and validation	Measures of Motivation for Psychiatric Treatment Based on Self- Determination Theory: Psychometric Properties in Dutch Psychiatric Outpatients	2014	348 patients with a primary diagnosis of mood, anxiety, psychotic, and personality disorders, Netherlands	HCCQ	To translate 3 measures based on SDT into Dutch and to investigate their psychometric properties in a population of patients with various primary psychiatric disorders in outpatient treatment
A6 (Juul, Maindal, Zoffmann, Frydenberg, & Sandbaek, 2011)	Quantitative—RCT—protocol	A cluster randomized pragmatic trial applying Self-determination theory to type 2 diabetes care in general practice	2011	40 general pratices and 4034 patients with diabetes, Denmark	HCCQ	To describe the design of a trial assessing the effectiveness of a training course for practice-nurses in autonomy support on patient-perceived motivation, HbA1, cholesterol, and well-being among a diabetes population, the actual intervention to a level of detail that allows its replication, and the connection between SDT recommendations for health care-provider behavior and the content of the training course
A7 (Kaap-Deeder et al, 2014)	Quantitative—RCT—protocol	Fostering Self- Endorsed Motivation to Change in Patients with an eating Disorder: The Role of Perceived Autonomy Support and Psychological Need Satisfaction	2014	84 patients with anorexia nervosa and bulimina nervosa, Belgium	HCCQ Perceived Parental Autonomy Support Scale (P-PASS)	To contribute to the existing literature on motivational dynamics in patients with eating disorders; To investigate the possible mean-level change in motivation;
						To examine the role of relative changes in psychological need satisfaction in relative changes in self-endorsed motivation;
A8 (Karlsen et al, 2016)	Mixed study—quasi-experimental design—protocol	Assessment of a web-based Guided Self-Determination Intervention for adults with type 2 diabetes in general practice: a study protocol	2016	172 patients with type 2 diabetes, Norway	HCCQ TSRQ	To assess the effectiveness of a web-based GSD programmed among adults with type 2 diabetes (T2DM) in general practice to improve diabetes self-management and glycosylated hemoglobin (HbA1c) through enhanced patient activation, self-management competence and autonomy
A9 (Lonsdale et al, 2012)	Quantitative—cluster RCT—protocol	Communication style and exercise compliance in physiotherapy (CONNECT). A cluster randomized controlled trial to teste a theory-based intervention to increase chronic low back pain patients’ adherence to physiotherapists’ recommendations: study rationale, design, and methods	2012	12 physiotherapists and 292 patients with chronic low back, Australia	HCCQ TSRQ	To assess the effect of an intervention designed to increase physiotherapists’ autonomy-supportive communication on low back pain patients’ adherence to physical activity and exercise therapy recommendations
A10 (Matthews et al, 2015)	Qualitative with focus group (1st phase) and interviews (2nd phase)	A brief on the development of a theoretically grounded intervention to promote patient autonomy and self-management of physiotherapy patients: face validity and feasibility of implementation	2015	9 physiotherapists, Ireland	HCCQ	To develop and pilot-test the feasibility of a theoretically derived implementation intervention to support physiotherapists in using an evidence-based autonomy supportive communication style in practice for promoting patient self-management in clinical practice
A11 (Rouse et al, 2014)	Quantitative—RCT—protocol	Fostering autonomous motivation, physical activity and cardiorespiratory fitness in rheumatoid arthritis: protocol and rationale for a randomized control trial	2014	100 participants with rheumatoid arthritis, United Kingdom (UK)	HCCQ	To target the key motivational processes underlying physical activity behavior change, with the intention of encouraging the adoption and maintenance of PA and in turn, improving cardiorespiratory fitness among rheumatoid arthritis patients
A12 Sripada, Bowersox, Ganoczy, Valenstein, & Pfeiffer, 2016)	Quantitative—correlational and cross-sectional	Self-Determination Theory and Outpatient Follow-Up After Psychiatric Hospitalization	2016	242 patients discharged from the inpatient psychiatric, USA	HCCQ TSRQ	To assess whether the constructs of self-determination theory—autonomy, competence, and relatedness—are associated with adherence to outpatient follow-up appointments after psychiatric hospitalization
A13 (Thomas, Wilson, Bedell, & Morse, 2019)	Qualitative—phenomenological	“They didn’t give up on me”: a women’s transitions clinic from the perspective of reentering women	2019	13 patients, of whom 11 had a substance use disorder, USA	HCCQ	To describes the experiences of women, including those with substance use histories, to inform our practice and that of others treating similar patients and conducted a process evaluation of our adapted Transitions Clinic Network model, informing how
A14 (Umeukeje et al, 2016)	Quantitative—multi-site cross-sectional	Health care providers’ support of patients’ autonomy, phosphate medication adherence, race and gender in end stage renal disease	2016	377 patients in dialysis, USA	HCCQ	best to meet the women’s practical, medical, and motivational needs To assess dialysis subjects’ perceived autonomy support association with phosphate binder medication adherence, race and gender
A15 (Halvari, Halvari, Bjørnebekk, & Deci, 2010)	Quantitative—scale validation	Motivation and anxiety for dental treatment: testing a self-determination theory model of oral self-care behavior and dental clinic attendance	2010	208 students, Norway	HCCQ	Relative autonomous motivation for dental treatment and perceived dental competence were expected to be positively associated with oral self-care
A16 (Halvari, Halvari, Bjørnebekk, & Deci, 2012)	Quantitative—correlational and cross-sectional	Motivation for Dental Home Care: Testing a Self-Determination Theory Model	2012	210 students, Norway	HCCQ	To better understand the issues related to dental clinic experiences, dental home care, and health
A17 (Halvari, Halvari, Bjørnebekk, & Deci, 2013)	Quantitative—correlational and cross-sectional	Oral health and dental well-being: testing a self-determination theory model	2013	208 students, Norway	HCCQ	To test if a Model SDT predicts oral health and dental well-being
A18 (Koponen, Simonsen, Laamanen, & Suominen, 2015)	Quantitative—correlational and cross-sectional	Health-care climate, perceived self-care competence, and glycémie control among patients with type 2 diabetes in primary care	2015	2866 patients with type 2 diabetes, Finland	HCCQ TSRQ	To examine whether the perceived health-care climate is associated with outcomes of care, in terms of perceived competence in diabetes care and glycémie control, when the effect of a wide variety of other important life-context is controlled for
A19 (Koponen, Simonsen, & Suominen, 2017b)	Quantitative—correlational and cross-sectional	Quality of primary health care and autonomous motivation for effective diabetes self-management among patients with type 2 diabetes	2017	2866 patients with type 2 diabetes, Finland	HCCQ TRSQ	To investigate whether, and how strongly, the 6 central quality dimensions of primary health care measured in this study (access to care, continuity of care, and autonomy support from one’s physician) are associated with autonomous motivation (self-regulation) for effective diabetes self-management among patients with type 2 diabetes
A20 (Koponen, Simonsen, & Suominen, 2018)	Quantitative—correlational and cross-sectional	Success in increasing physical activity (PA) among patients with type 2 diabetes: a self-determination theory perspective	2018	1256 patients with type 2 diabetes, Finland	HCCQ TRSQ	To identify factors that predict success in increasing physical activity among patients with type 2 diabetes
A21 (Koponen, Simonsen, & Suominen, 2019)	Quantitative—correlational and cross-sectional	How to promote fruits, vegetables, and berries intake among patients with type 2 diabetes in primary care? A self-determination theory perspective	2019	2866 patients with type 2 diabetes, Finland	HCCQ TRSQ	To investigate a whether perceived autonomy support (from a physician), autonomous motivation, and self-care competence were associated with fruits, vegetables, and berries intake (FVBI) among patients with type 2 diabetes when the effects of other important life-.context factors (perceived health, medication, duration of diabetes, mental health, stress, and social support) were controlled for and whether autonomous motivation and self-care competence mediated the effect of perceived autonomy support on FVBI
A22 (Martin, Byrd, Wooster, & Kulik, 2017)	Quantitative—correlational and cross-sectional	Self-determination theory: the role of the health care professional in promoting mindfulness and perceived competence	2017	131 students, USA	HCCQ	To predict mindfulness and perceived competence using self-determination theory (SDT)
A23 (Münster Halvari, Halvari, & Deci, 2018)	Quantitative—correlational and cross-sectional	Attending and avoiding dental appointments: Do “bright” and “dark” motivational paths have a role?	2017	322 students, Norway	HCCQ TRSQ	To test a self-determination theory (SDT) process model of the “bright” and the “dark” motivational pathways through dental attendance or avoidance to oral health
A24 (Koponen, Simonsen, & Suominen, 2017a)	Quantitative—correlational and cross-sectional	Determinants of physical activity among patients with type 2 diabetes: the role of perceived autonomy support, autonomous motivation and self-care competence	2016	2866 patients with type 2 diabetes, Finland	HCCQ TRSQ	To investigate, whether the 3 central SDT variables (perceived autonomy support, autonomous motivation and self-care competence), were associated with engagement in physical activity among patients with type 2 diabetes when the effect of a wide variety of other important life context factors (perceived health, medication, duration of diabetes, mental health, stress and social support) was controlled for
A25 (Murray et al, 2019)	Quantitative—correlational and cross-sectional	Assessing physiotherapists’ patient autonomy for self-management: reliability and validity of the communication evaluation in rehabilitation tool	2018	24 physiotherapists and 24 patients with chronic low back pain, Ireland	HCCQ	To assess the inter-rater reliability and concurrent validity of the Communication Evaluation in Rehabilitation Tool, which aims to externally assess physiotherapists competency in using Self-Determination Theory-based communication strategies in practice
A26 (Vian et al, 2018)	Quantitative—RCT	The role of motivation antiretroviral therapy adherence in China	2018	115 patients with HIV/AIDS, USA	HCCQ TSRQ	To asses show components of the SDT model changed in response to an intervention to increase medication adherence and to explore the relative importance of autonomous and controlled forms of motivation
A27 (Yu et al, 2015)	Quantitative—correlational and cross-sectional	Motivation-related predictors of physical activity engagement and vitality in rheumatoid arthritis patients	2015	335 patients with rheumatoid arthritis, UK	HCCQ	To test, in a sample of rheumatoid arthritis patients, Basic Psychological Needs Theory-based hypothesized motivational sequence (autonomy support to basic need satisfaction to motivation regulations to PA/well-being)
A28 (Fu et al, 2019)	Q uantitative—crossover randomized design	Influence of Patient Characteristics and Psychological Needs on Diabetes Mobile App Usability in Adults with Type 1 or Type 2 Diabetes: Crossover randomized Trial	2019	92 patients with type 1 or type 2 diabetes, USA	HCCQ	To assess the effect of patient characteristics on app usability, and determine whether patient characteristics and psychological needs (competence, autonomy, and connectivity) important for motivation in diabetes care are associated with app usability
A29 (Zuroff, Koestner, Moskowitz, McBride, & Bagby, 2012)	Quantitative—RCT	Therapist’s Autonomy support and Patient’s Self-Criticism Predict Motivation During Brief Treatments for Depression	2012	95 patients with depression, Canada	HCCQ TSRQ	To investigate predictors of relapse in depressed outpatients who were first successfully treated with cognitive behavioral therapy, interpersonal therapy or pharmacotherapy with clinical management
A30 (Quinlivan, Messer, Roytburd, & Blickman, 2017)	Quantitative—correlational and cross-sectional	Unmet Core Needs for Self-Determination in HIV-infected Women of Color in Medical Care	2017	189 patients with HIV, USA	Basic Needs Satisfaction in General Scale (BNSG)	To identify the distribution of relatedness, autonomy, and competence achievement among a sample of HIV-infected women of care and explore the demographic and health-related factors that appear associated with these precursors for intrinsic-motivation development
A31 (Taylor, Piatt, Hill, & Malcolm, 2012)	Quantitative—case-control study	Diabetes camps and Self-Determination Theory: Controlling Glycémie Level in Youth with Type 1 Diabetes	2012	10 patients with type 1 diabetes and 11 parents, USA	HCCQ	To examine the effects of attending a medical specialty diabetes camp on perception of autonomy support and management of type 2 diabetes among campers and parents
A32 (Boiché, Gourlan, & Rubin, 2018)	Quantitative—RCT	Impact of a residential program on the psychological needs, motivation and physical activity of obese adults: A controlled trial based on Self-Determination Theory	2018	49 patients obese, France	BNSG	To examine the increased benefits of a SDT-based motivation component on psychological needs’ fulfillment, self-determined motivation and physical activity of obese taking part in a rehabilitation program
A33 (van der Zee, Baars-Elsinga, Visser-Meily, & Post, 2013)	Quantitative—correlational and cross-sectional	Responsiveness of 2 participation measures in an outpatient rehabilitation setting	2013	69 patients with brain injury or neuromuscular disease, Netherlands	Impact on participation and autonomy (IPA)	To compare the responsiveness of the IPA and the USER-Participation in patients who followed an outpatient rehabilitation programme and to examine the concurrent validity of the USER- Participation as compared with the IPA
A34 (Hoseynrezaee, Kordikarimabadi, & Jahani, 2017)	Quantitative—correlational and cross-sectional	Attitudes of Patients with Cancer Towards Truth-telling and Self-Determination in Kerman, 2016: a Crosse Sectional Study	2017	214 patients with cancer, Iran	Autonomy Preference Index (API)	To investigate the attitudes of Iranian patients with cancer towards truth-telling and self-determination

The articles were categorized according to the methodological paradigm of the study. Thus, we found 16 quantitative, correlational and cross-sectorial study,^18–33^ 9 quantitative, randomized control trial study,^34–42^ 1 quantitative, correlational and longitudinal study,^43^ 1 quantitative, scale translation and validation,^44^ 1 mixed study, quasi-experimental design protocol study,^45^ 1 qualitative with focus group (1^st^ phase) and interviews (2^nd^ phase),^46^ 1 qualitative, multi-site cross-sectional study,^47^ 1 quantitative, scale validation,^48^ 1 quantitative, randomized crossover design^49^ and 1 quantitative case-control study.^50^ Eight of these studies were carried out in the United States of America (USA),^19,26,31,40,47,50–52^ 7 in Norway,^20,21,27,34,43,45,48^ 5 in Finland,^22–25,28^ 3 in Holland,^32,35,44^ 2 in England,^30,39^ 1 in Australia,^38^ 1 in Thailand,^18^ 1 in Denmark,^36^ 1 in Belgium,^37^ 1 in Ireland,^29,46^ 1 in Canada,^53^ 1 in France^54^ and 1 in Iran.^33^

**Figure 2:**
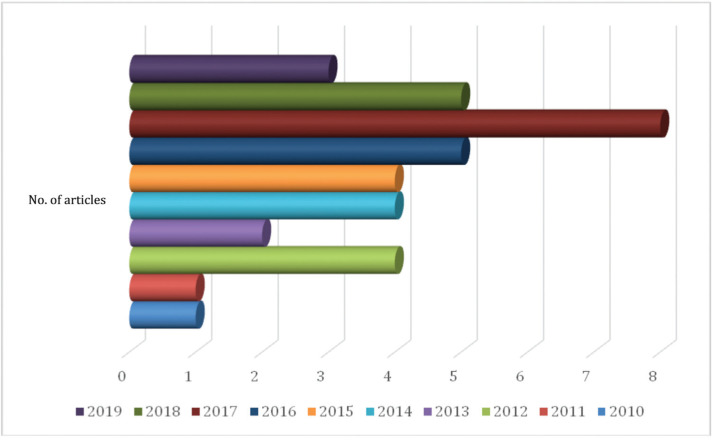
Distribution of studies, according to the year of publication, Porto, Portugal, 2020.

Regarding the year of publication of the articles, in Figure 2, we observe the distribution by year of publication. It is possible to observe that the research refers to the period between the years 2010 and 2020. Among the 10 years included in the research, 2017 is highlighted with 8 articles.

In total 34 publications, it was possible to identify 7 different instruments, namely: The Health Care Climate Questionnaire (HCCQ),^18–30,35–40,43–48,50–53^ Treatment Self-Regulation Scale (TRSQ),^19,22–25,27,28,34,38,40,43,45,53^ Basic Needs Satisfaction in General Scale (BNSG),^31,54^ Impact Participation and Autonomy (IPA),^32^ Autonomy Preference Index (API)^33^ and Perceived Parental Autonomy Support Scale (P-PASS).^37^

The HCCQ has a long-form that presents 15 items and another short form with 6 items (including items 1, 2, 4, 7, 10 and 14, from the full scale). For each of the items, the Likert scale is used from 1 to 7, in which 1 is used to state that “it is not true” and 7 is “very true”. The total score should be calculated by adding the items on the scale; however, in the extended version, item 13 is inverted. Higher scores represent an increasingly higher level of support for perceived autonomy.^55^ Specifically, depending on the study question, the HCCQ can be used to evaluate the patients’ perceptions of the degree to which health professionals support their autonomy, or it can be used to assess patients’ perceptions of the degree to which the multidisciplinary team is autonomous.^55^ From the research carried out, it appears that this is the most used instrument to assess autonomy, being used in 29, among the 34 selected studies.^18–30,35–40,43–48,50–53^

The TRSQ quiz evaluates individual-specific differences concerning the domains of motivation and regulation, that is, the questions concern the regulation of a specific behavior or the class of behaviors. This instrument is used to understand why people try to change behavior and follow treatment. Some different versions of the questionnaire can be found; however, each version assesses the extent to which the person’s motivation for health behaviors is autonomous. In the majority of the versions, there are 2 subscales: autonomous regulation and controlled regulation. The quantity of items, in its turn, differs according to what you want to study. For example, the version that referred to the health behaviors of the patients with diabetes has 19 items, the version that intends to evaluate participation in a weight loss program with reduced calorie consumption and medical supervision has 18 items, and the shorter version with 13 items can also be used. In each of the items, the responses comprise a Likert scale from 1 to 7, where 1 refers to the answer “it is not true” and 7 to the answer “it is very true” .^55^ The present study concludes that, between the 34 articles, thirteen uses this instrument to evaluate the autonomous behaviors.^19,22–25,27,28,34,38,40,43,45,53^

The P-PASS is a scale with 24 questions that aim to assess the parent’s behavior supporting children’s autonomy. This scale assesses the following behaviors: choose within certain limits, recognize feelings, threats of punishment, the pressure to perform tasks and criticisms that develop feelings of guilt. The answers are given in a Likert scale from 1 to 7, where 1 refers to the answer “I don’t agree at all” and 7 to the answer “accepts very strongly”.^55^ In this research, only one study uses this instrument.^37^

The Basic Needs Satisfaction in General Scale (BNSG) is a 21 items scale that assesses the autonomy, the relationship and the competence associated with self-determination. This allows assessing the participants’ perceptions of autonomy (5 items, for example, “I generally feel free to express my ideas and options”), competency (5 items, for example, “I have the impression that I am doing what is right”) and the relationship (5 items like “I feel comfortable with other people”). The response options range from 1 to 7, with 1 being assigned to “never true” and 7 to “very true”. The autonomy scale includes 7 items, and the relationship contains 8, and the competency includes 6 items. Items with a negative structure should be encoded inversely.^56,57^ In the present study, only 2 studies use this instrument.^31,54^

The IPA questionnaire consists of questions about daily life activities, more specifically, 39 questions in 5 domains: internal Autonomy, external Autonomy, family roles, social and work relationships and paid education. The answers comprise a Likert scale ranging from 0, corresponding to “very good” to 4, identifying “very poor”. The average of the scores related to each domain is calculated to obtain an overall score for that domain.^58^ In the present study, only one study used this instrument.^32^ Its application aims to obtain patients’ opinions on how their health condition or disability affects their ability to live the life they want and evaluate their participation in activities.

The API was developed to evaluate the patient preferences about information and the involvement in medical decisions. Its French version has 23 items, which can be classified from 0 “totally disagree” and 4 “totally agree”. Among these 23 items, 8 refer to the preference for information and fifteen to the preference for participation (6 items assess the general preference for participations and 9 assess the preference for participation in terms of diseases of different severities).^59^ Of the consulted studies that resulted from the research carried out, only one study used this instrument.^33^

## Discussion

The instruments to measure human needs have constituted, in the practice of care, essential tools that allow health professionals to realize effective health gains.^60^

The HCCQ was designed to be used by the patients, allowing them to report their perceptions relative to how health professionals support their autonomy. Thus, this instrument is based on the patient’s perceptions of how they relate to health professionals, respect for informed consent and health empowerment. In this context, the importance of the empathic relationship in the relation of the person’s autonomy is noted.^55^ In the present study, it appears that all studies that use this instrument intend to understand the way the patient interprets and reacts to the support provided by health professionals, namely doctors, nurses, dentists, physiotherapists, among other professionals.^18–30,35–40,43–48,50–53^ If the perceived autonomy is related to the satisfaction of their basic needs and if it influences their quality of life.^18^

It should be noted that all studies using this instrument are based on the theory of self-determination by Deci and Ryan. In addition to autonomy, this theory assesses competence and relationship, emphasizing that these are 3 individual psychological needs.^61^ Competence, in turn, consists of the need to feel effective in the actions you aspire to and perform. Finally, a relationship is the desire to feel connected with others, with the society around us, to belong and to be valued.^61^

This instrument was used in qualitative with focus group (1st phase) and interviews (2nd phase), and phenomenological studies,^46,51^ quantitative, correlacional, cross-sectorial or longitudinal studies,^18–30,43,47^ in quantitative randomized control trial studies,^35–40,52,53^ in mixed, quasi-experimental design studies,^45^ in quantitative, case-control studies^50^ and in studies that aimed at translating and validating the instrument into other languages.^44,48^

The TSRQ assesses a person’s degree of motivation for health behaviors, and whether they are autonomous, depending on the behavior that is intended to be studied. The scale can be used to assess this motivation for various behaviors, such as the version referring to patients’ behaviors with diabetes and the version referring to participation in a weight loss program. Several versions were used by the studies found in the present research, namely the version of the autonomous motivation for the control of diabetes,^22,24,25,34,45^ for adherence to a rehabilitation program,^43^ for adherence to the practice of physical exercise and rehabilitation plan,^23,28,38^ for adherence to consultations after psychiatric hospitalisation,^19^ for adherence to oral health programs,^27^ for adherence to drug treatment^40^ and, finally, for adherence to cognitive-behavioural therapy, interpersonal therapy and pharmacotherapy.^53^

This instrument allows, in this way, to evaluate the reasons why the person took a particular behavior and to verify if that decision making was autonomous and without the influence of third parties and was used in quantitative, correlacional, cross-sectorial or longitudinal studies,^19,22–25,27,28,43^ in mixed, quasi-experimental design^45^ and quantitative randomized control trial.^34,38,40,53^

According to the theory of self-determination, the BNSG scale was designed to assess autonomy, competence, and relationship.^57,62^ Regarding autonomy, this consists of 7 statements. Expressions such as “I feel I am free to decide how to live my life”, “I feel pressured in my life”, “I generally feel to express my ideas and opinions”, “I often have to do what they tell me”, “I interact daily and tend to take my feelings into account”, “I feel like I can practically be myself in my daily situations” and “I don’t have many opportunities to decide for myself how to do things in my daily life activities“.^57,62^ These expressions that evaluate autonomy show the relevance that the authors attribute to decision-making. This scale referred to in the present research was used in 2 studies.^31,54^

The IPA has the sub-scales of internal and external autonomy, family roles, social relations and questions about the development of work and education.^58^ This scale is used to assess the daily life activities related to the patient’s ability to move around, the capacity for self-care, taking into account whether or not he can decide when and how he wants to perform these activities. It also takes into account the patient’s ability to carry out activities inside and outside the home, to take care of his finances, to promote moments of leisure, as well as his social life and relationships, the support and help of other people, and the possibility finding or maintaining paid or voluntary employment. All of these activities should also be evaluated against the help of third parties.^58^ The IPA was designed to assess the impact of illness or diseases on the described life activities and. Therefore, it is applied in situations of illness.

The API aims to assess the patient’s participation and desires for information in health-related decisions. The 23 questions refer to decision-making and informed consent.^59^ In the current research, this index was applied by only one study.^33^

The Perceived Parental Autonomy Support Scale is used to find out what the child’s perception of the autonomy offered by their parents or their substitutes, evaluating how they allow them to make decisions within certain limits if they explain the reasons behind the requirements, rules and limits imposed and whether the parents are aware, accept and recognise the child’s feelings.^63^

### Instruments limitations

The usability of the majority of the identified instruments was particularly studied and adapted, mainly because the HCCQ allows a readjustment, for the possibility of utilization of the short version,^18–30,35–40,43–48,50–53^ as well as the TSRS for the various versions, which allow the assessment of healthy health behaviors.^19,22–25,27,28,34,38,40,45,53^

Although the articles’ methodological quality has not been analyzed, there was a considerable disparity in these articles’ format and content, as they adapted the instruments according to the objectives, sometimes using the shorter versions of the instruments.

Of these instruments found in the research, we found out that the majority report only to evaluating perceptions about respect for decision-making and informed consent.^18–40,43–48,50–54^

We recall that the guiding principle for defining the inclusion criteria was to identify a minimum set of the necessary information that: address the person (participants), address the autonomy (concept), and use instruments to assess the autonomy (context), for possible analysis.

The reasons for the existence of limited evidence regarding instruments that allow measuring autonomy can be very complex, since the concept of autonomy is, in itself, a multidimensional concept, and, in practice, it is easier to analyze 1 or 2 of these dimensions.^1^ It should be noted that none of the instruments assesses emotional management capacity, as well as cognitive capacity, intellectual capacity, physical capacity, and the capacity for social interaction. There are, therefore, questions that arise. Can we consider an autonomous person, just because he makes decisions without the influence of others? Does this person have the relevant information for that autonomous decision-making? Concerning emotional management, is this person in the best time to make autonomous decisions regarding essential aspects of his life?

The truth is that the health professionals that work directly with the need for autonomous management need instruments that can measure in an effective way autonomy, without neglecting any of the aspects addressed. To make autonomous decisions, a person will have to meet the minimum conditions for this autonomy exercise, such as managing emotions effectively, being socially integrated, and having the cognitive and intellectual capacity and physical condition to put his decisions into practice.

## Conclusion

This review’s results provide a substantial contribution to systematize the available instruments that allow assessing the person’s autonomy, since its assessment and monitoring is essential to guarantee its exercise. In clinical practice, the importance of maintaining/promoting autonomy cannot be omitted because it has direct repercussions on the person’s quality of life and dignity. However, there are many limitations in the instruments used for its evaluation, since all of them refer only to empowerment and informed consent. Hence, it is necessary to develop instruments that can measure it, guided by objectives that meet the evaluation of dimensions such as: emotional management capacity, physical capacity, cognitive capacity and social integration. Although 3 scales (Impact Participation and Autonomy, Psychological needs and Basic Needs Satisfaction in General Scale) highlight the social relationship, it is not understood as necessary to express autonomy, constituting different sub-scales.

Some assessment tools, such as the Health Care Climate Questionnaire, a Treatment Self-Regulation Scale, Basic Needs Satisfaction in General Scale, Psychological needs, Impact Participation and Autonomy, and Autonomy Preference Index, can be used by nurses to promote autonomy.

It should be noted that the results show the need for more significant investment at this level so that it is possible to develop instruments that allow facing the limitations of the instruments currently used.

Future studies should consider the consensus on the construction of an instrument related to aspects that promote autonomy, such as, for example, using a Delphi Method, drawing on the reflection of experts on the subject, which may generate more knowledge.
